# Comparing 2 crystal structures and 12 AlphaFold2-predicted human membrane glucose transporters and their water-soluble glutamine, threonine and tyrosine variants – CORRIGENDUM

**DOI:** 10.1017/qrd.2022.8

**Published:** 2022-07-21

**Authors:** Eva Smorodina, Fei Tao, Rui Qing, David Jin, Steve Yang, Shuguang Zhang

In the aforementioned article, the authors’ conflict of interest statement is incorrectly worded. The statement should read: *The funder Avalon Globo-Care Corp had no interference in the design of the study; in the collection, analyses, or interpretation of data; in the writing of the manuscript, or in the decision to publish the results.* The authors apologise for this error.
