# Supplementary material for: Comparing 2 crystal structures and 12 AlphaFold2-predicted human membrane glucose transporters and their water-soluble glutamine, threonine and tyrosine variants
Source: QRB Discov. 2022 Jun 13;3:e5. doi: 10.1017/qrd.2022.6 (PMC10392618; doi:10.1017/qrd.2022.6)
Supplement: Supplementary file 1 [file S2633289222000060sup001.docx]

**Comparing 2 crystal structures and 12 AlphaFold2-predicted human membrane glucose transporters and their water-soluble QTY variants**

Eva Smorodina^1^, Fei Tao^2^, Rui Qing^2^, David Jin^3^, Steve Yang^4^ and Shuguang Zhang^5^*

^1^Laboratory for Computational and Systems Immunology, Department of Immunology, University of Oslo, Norway

^2^Laboratory of Food Microbial Technology, State Key Laboratory of Microbial Metabolism, School of Life Sciences and Biotechnology, Shanghai Jiaotong University, Shanghai 200240, China.

^3^Avalon GloboCare Corp. Freehold, New Jersey 07728, USA

^4^PT Metiska Farma, Daerah Khusus Ibukota Jakarta 12220, Indonesia

^5^Laboratory of Molecular Architecture, Media Lab, Massachusetts Institute of Technology, 77 Massachusetts Avenue, Cambridge, MA, 02139, USA

*To whom the correspondence should be addressed.

Email: [Shuguang@MIT.EDU](mailto:Shuguang@MIT.EDU)

Telephone: +1-617-258-7514

Eva Smorodina, [ribes.ev@gmail.com](mailto:ribes.ev@gmail.com), ORCID: [0000-0002-5457-5163](https://orcid.org/0000-0002-5457-5163)

Fei Tao [taofei@sjtu.edu.cn](mailto:taofei@sjtu.edu.cn), ORCID: [0000-0002-5997-8770](https://orcid.org/0000-0002-5997-8770)

Rui Qing, [ruiqing.br@sjtu.edu.cn](mailto:ruiqing.br@sjtu.edu.cn), ORCID: [0000-0002-7952-2295](https://orcid.org/0000-0002-7952-2295)

David Jin [david@avalon-globocare.com](mailto:david@avalon-globocare.com), ORCID: [0000-0002-3061-4228](https://orcid.org/0000-0002-3061-4228)

Steve Yang, [steve.yang@metiska.co.id](mailto:steve.yang@metiska.co.id), ORCID: [0000-0002-5346-0321](https://orcid.org/0000-0002-5346-0321)

Shuguang Zhang, [Shuguang@MIT.EDU](mailto:Shuguang@MIT.EDU), ORCID: [0000-0002-3856-3752](https://orcid.org/0000-0002-3856-3752)

**Supplementary Materials**

**Table S1. Prediction confidence for GLUT1^native^, GLUT1^QTY^, GLUT3^native^, and GLUT3^QTY^.** “Protein” column represents native and QTY versions of GLUT1 and GLUT3. “Model” column defines the model ID (PDB-files of all models have the names as mentioned in this column). “Rank” column shows how good the model is (the better pLDDT score is, the higher the model rank is). Models are sorted by their rank. We use the models with the highest rank (rank 1). Confidence in models based on pLDDT values: very low (pLDDT < 50), low (pLDDT = 60), OK (pLDDT = 70), confident (pLDDT = 80), very high (pLDDT > 90).

| Protein | Model | Rank | pLDDT | pTMscore |
| --- | --- | --- | --- | --- |
| GLUT1 native | 3 | 1 | 89.45 | 0.8859 |
| GLUT1 native | 5 | 2 | 89.31 | 0.8937 |
| GLUT1 native | 2 | 3 | 87.44 | 0.8507 |
| GLUT1 native | 4 | 4 | 86.64 | 0.8632 |
| GLUT1 native | 1 | 5 | 86.54 | 0.8383 |
| GLUT1 QTY | 3 | 1 | 88.24 | 0.8670 |
| GLUT1 QTY | 5 | 2 | 87.45 | 0.8716 |
| GLUT1 QTY | 1 | 3 | 86.91 | 0.8516 |
| GLUT1 QTY | 2 | 4 | 86.15 | 0.8399 |
| GLUT1 QTY | 4 | 5 | 86.12 | 0.8523 |
| GLUT3 native | 5 | 1 | 89.71 | 0.8972 |
| GLUT3 native | 3 | 2 | 88.69 | 0.8849 |
| GLUT3 native | 1 | 3 | 87.39 | 0.8445 |
| GLUT3 native | 2 | 4 | 86.87 | 0.8481 |
| GLUT3 native | 4 | 5 | 86.50 | 0.8726 |
| GLUT3 QTY | 3 | 1 | 86.42 | 0.8573 |
| GLUT3 QTY | 1 | 2 | 86.32 | 0.8407 |
| GLUT3 QTY | 5 | 3 | 84.97 | 0.8528 |
| GLUT3 QTY | 2 | 4 | 83.27 | 0.8089 |
| GLUT3 QTY | 4 | 5 | 81.34 | 0.7756 |

**Table S2. Prediction confidence for native glucose transporters, GLUT2^QTY^, and GLUT4^QTY^-14^QTY^.** “Protein” column represents native and QTY versions of GLUT2 and GLUT4-14. “Model” column defines the model ID (PDB-files of all models have the names as mentioned in this column). Confidence in models based on pLDDT values: very low (pLDDT < 50), low (pLDDT = 60), OK (pLDDT = 70), confident (pLDDT = 80), very high (pLDDT > 90). You can find information about other models in the GitHub Repository: <https://github.com/eva-smorodina/glucose-transporters>.

| **Protein** | **Model** | **pLDDT** | **pTMscore** |
| --- | --- | --- | --- |
| GLUT2 native | 5 | 86.01 | 0.8658 |
| GLUT2 QTY | 3 | 85.01 | 0.8256 |
| GLUT4 native | 5 | 87.86 | 0.8770 |
| GLUT4 QTY | 3 | 84.95 | 0.8423 |
| GLUT5 native | 3 | 89.39 | 0.8960 |
| GLUT5 QTY | 3 | 87.63 | 0.8799 |
| GLUT6 native | 3 | 82.12 | 0.8231 |
| GLUT6 QTY | 3 | 82.03 | 0.8173 |
| GLUT7 native | 3 | 87.99 | 0.8655 |
| GLUT7 QTY | 3 | 86.84 | 0.8513 |
| GLUT8 native | 3 | 81.88 | 0.8293 |
| GLUT8 QTY | 3 | 83.85 | 0.8365 |
| GLUT9 native | 1 | 83.30 | 0.8137 |
| GLUT9 QTY | 1 | 81.94 | 0.8009 |
| GLUT10 native | 3 | 74.64 | 0.7586 |
| GLUT10 QTY | 3 | 74.08 | 0.7429 |
| GLUT11 native | 3 | 88.51 | 0.8772 |
| GLUT11 QTY | 3 | 87.85 | 0.8665 |
| GLUT12 native | 3 | 73.04 | 0.7404 |
| GLUT12 QTY | 1 | 72.98 | 0.7193 |
| GLUT13 native | 3 | 80.02 | 0.7751 |
| GLUT13 QTY | 3 | 79.70 | 0.7733 |
| GLUT14 native | 5 | 87.30 | 0.8700 |
| GLUT14 QTY | 3 | 84.25 | 0.8344 |

**Figure S1. 14 human glucose transporter and their water-soluble QTY variants:**

**protein sequences, predicted 2D structures, and hydrophobic profiles**

GTR1=GLUT1=SLC2A1= facilitated glucose transporter member 1, [P11166](https://www.uniprot.org/uniprot/P11166), 492aa


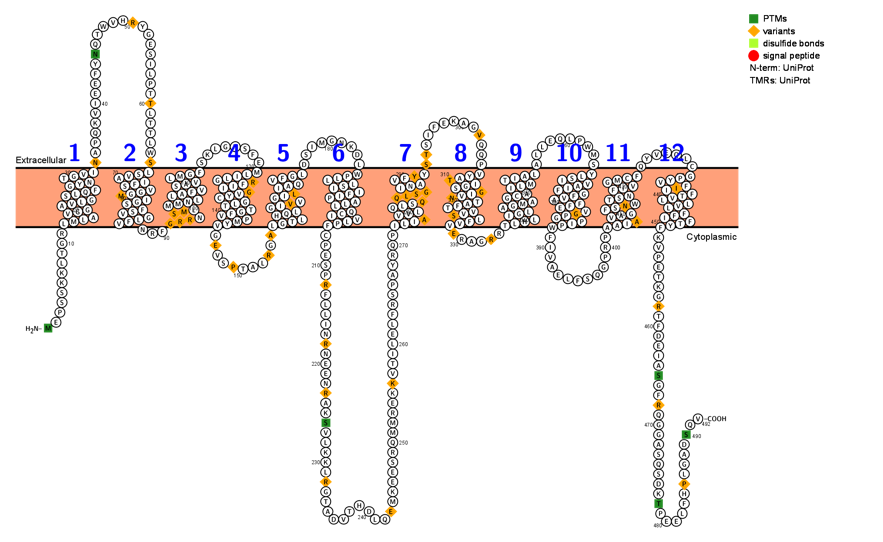

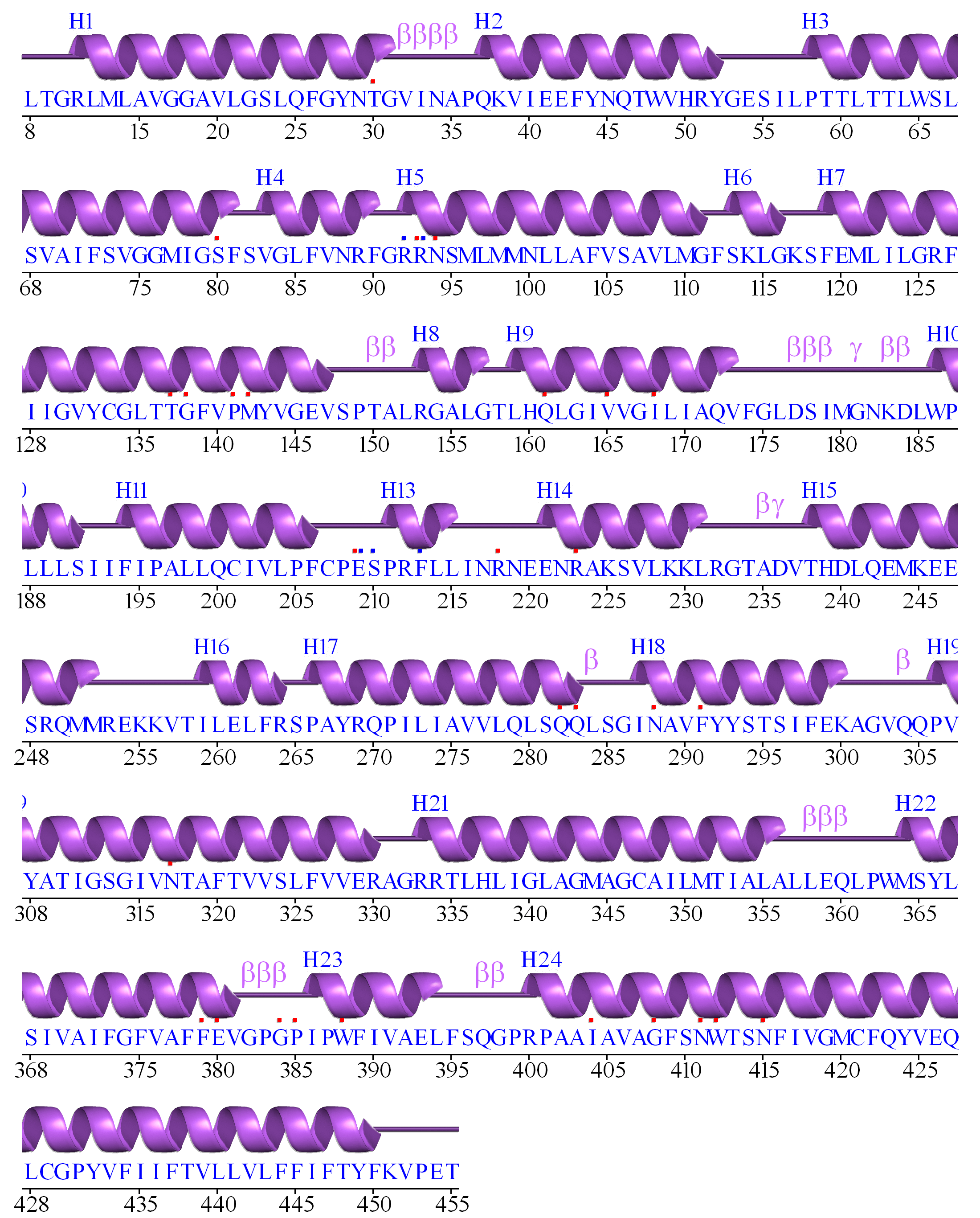


>sp|P11166| GTR1=GLUT1 Solute carrier family 2, facilitated glucose transporter member 1

MEPSSKKLTGRLMLAVGGAVLGSLQFGYNTGVINAPQKVIEEFYNQTWVHRYGESILPTT

LTTLWSLSVAIFSVGGMIGSFSVGLFVNRFGRRNSMLMMNLLAFVSAVLMGFSKLGKSFE

MLILGRFIIGVYCGLTTGFVPMYVGEVSPTALRGALGTLHQLGIVVGILIAQVFGLDSIM

GNKDLWPLLLSIIFIPALLQCIVLPFCPESPRFLLINRNEENRAKSVLKKLRGTADVTHD

LQEMKEESRQMMREKKVTILELFRSPAYRQPILIAVVLQLSQQLSGINAVFYYSTSIFEK

AGVQQPVYATIGSGIVNTAFTVVSLFVVERAGRRTLHLIGLAGMAGCAILMTIALALLEQ

LPWMSYLSIVAIFGFVAFFEVGPGPIPWFIVAELFSQGPRPAAIAVAGFSNWTSNFIVGM

CFQYVEQLCGPYVFIIFTVLLVLFFIFTYFKVPETKGRTFDEIASGFRQGGASQSDKTPE

ELFHPLGADSQV

The QTY variant GTR1^QTY^

MEPSSKKLTGRQMQATGGATQGSQQFGYNTGTTNAPQKVIEEFYNQTWVHRYGESILPTT

LTTLWSQSTATYSTGGMTGSYSTGQYTNRFGRRNSMQMMNQQAYTSATQMGYSKLGKSFE

MQQQGRYTTGTYCGQTTGYTPMYTGEVSPTALRGAQGTQHQQGTTTGTQTAQTYGQDSIM

GNKDQWPQQQSTTYTPAQQQCTTQPYCPESPRFLLINRNEENRAKSVLKKLRGTADVTHD

LQEMKEESRQMMREKKVTILELFRSPAYRQPTQTATTQQQSQQQSGTNATYYYSTSIFEK

AGVQQPTYATTGSGTTNTAYTTTSQYTTERAGRRTQHQTGQAGMAGCATQMTTAQAQQEQ

LPWMSYQSTTATYGYTAYYETGPGPTPWYTTAELFSQGPRPAATATAGYSNWTSNYTTGM

CYQYVEQLCGPYTYTTYTTQQTQYYTYTYYKVPETKGRTFDEIASGFRQGGASQSDKTPE

ELFHPLGADSQV


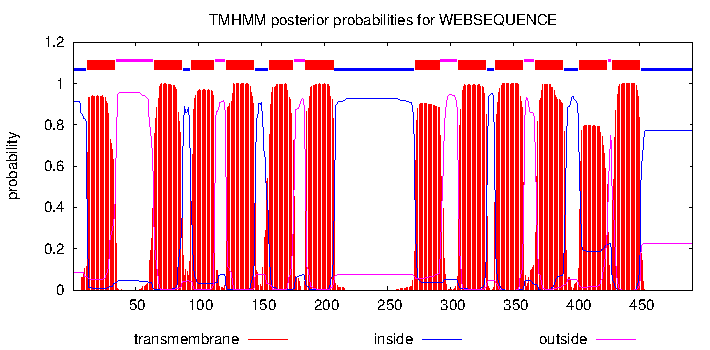

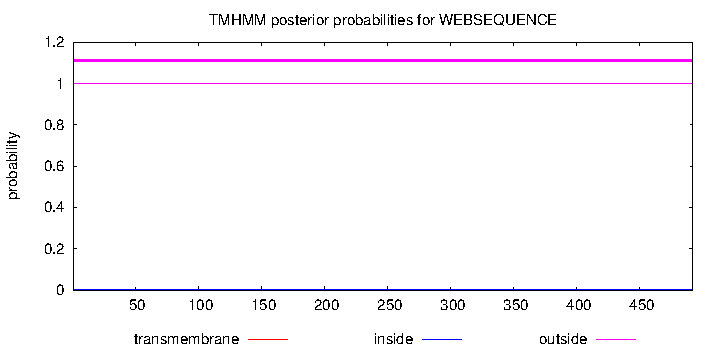


Native GTR1 GTR1^QTY^

GTR2=Glut2=SLC2A2= facilitated glucose transporter member 2, [P11168](https://www.uniprot.org/uniprot/P11168), 524aa


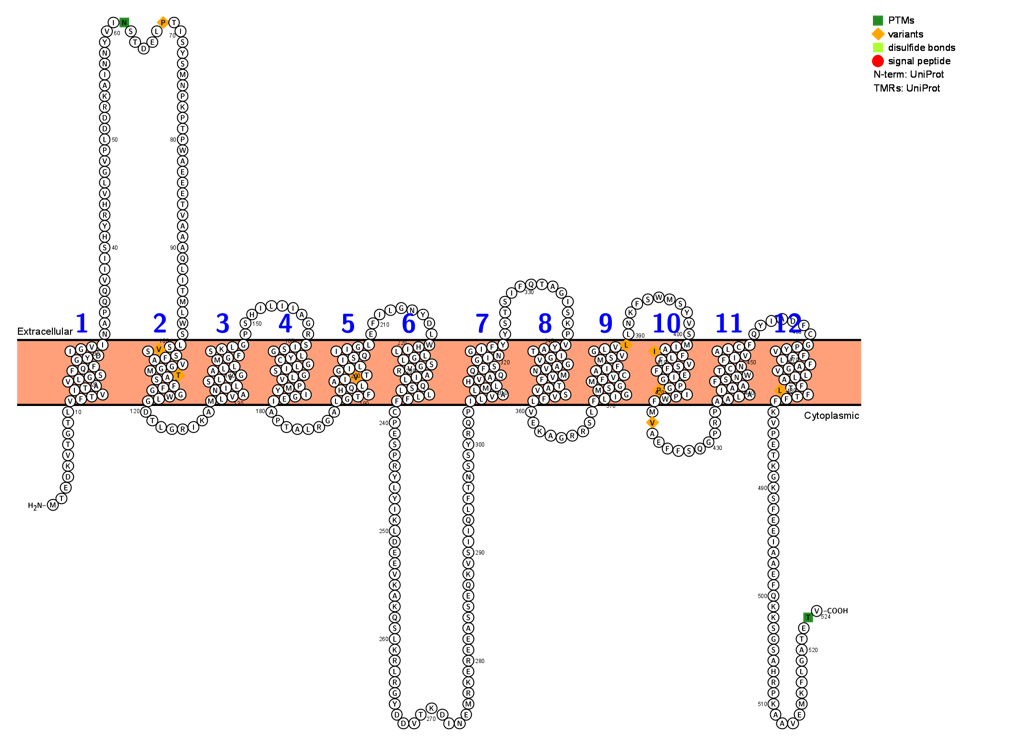


[P11168](https://www.uniprot.org/uniprot/P11168)|GTR2 Solute carrier family 2, facilitated glucose transporter member 2

GTR2=GLUT2

MTEDKVTGTLVFTVITAVLGSFQFGYDIGVINAPQQVIISHYRHVLGVPLDDRKAINNYV

INSTDELPTISYSMNPKPTPWAEEETVAAAQLITMLWSLSVSSFAVGGMTASFFGGWLGD

TLGRIKAMLVANILSLVGALLMGFSKLGPSHILIIAGRSISGLYCGLISGLVPMYIGEIA

PTALRGALGTFHQLAIVTGILISQIIGLEFILGNYDLWHILLGLSGVRAILQSLLLFFCP

ESPRYLYIKLDEEVKAKQSLKRLRGYDDVTKDINEMRKEREEASSEQKVSIIQLFTNSSY

RQPILVALMLHVAQQFSGINGIFYYSTSIFQTAGISKPVYATIGVGAVNMVFTAVSVFLV

EKAGRRSLFLIGMSGMFVCAIFMSVGLVLLNKFSWMSYVSMIAIFLFVSFFEIGPGPIPW

FMVAEFFSQGPRPAALAIAAFSNWTCNFIVALCFQYIADFCGPYVFFLFAGVLLAFTLFT

FFKVPETKGKSFEEIAAEFQKKSGSAHRPKAAVEMKFLGATETV


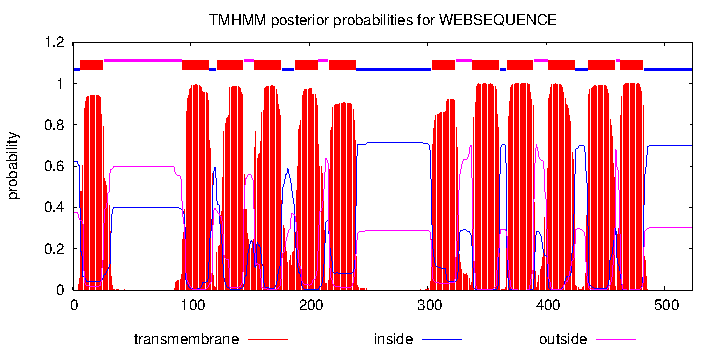

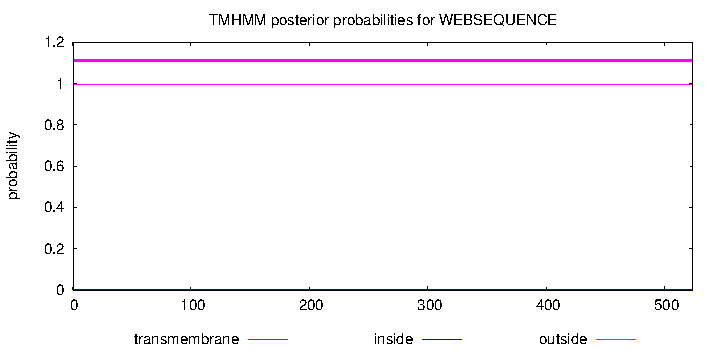


GLUT2^QTY^

MTEDKVTGTLTYTTTTATQGSYQYGYDTGTTNAPQQVIISHYRHVLGVPLDDRKAINNYV

INSTDELPTISYSMNPKPTPWAEEETVAAAQLITMLWSQSTSSYATGGMTASYYGGWQGD

TLGRIKAMQTANTQSQTGAQQMGYSKQGPSHILIIAGRSTSGQYCGQTSGQTPMYTGETA

PTALRGAQGTYHQQATTTGTQTSQTTGQEFILGNYDQWHTQQGQSGTRATQQSQQQYYCP

ESPRYLYIKLDEEVKAKQSLKRLRGYDDVTKDINEMRKEREEASSEQKVSIIQLFTNSSY

RQPTQTAQMQHTAQQYSGTNGTYYYSTSIFQTAGISKPTYATTGTGATNMTYTATSTYQT

EKAGRRSLYQTGMSGMYTCATYMSTGQTQQNKFSWMSYVSMTATYQYTSYYETGPGPTPW

YMVAEFFSQGPRPAAQATAAYSNWTCNYTTAQCYQYIADFCGPYTYYQYAGTQQAYTQYT

YYKVPETKGKSFEEIAAEFQKKSGSAHRPKAAVEMKFLGATETV

GTR3=GLUT3=SLC2A3= facilitated glucose transporter member 3, [P11169](https://www.uniprot.org/uniprot/P11169), 496aa


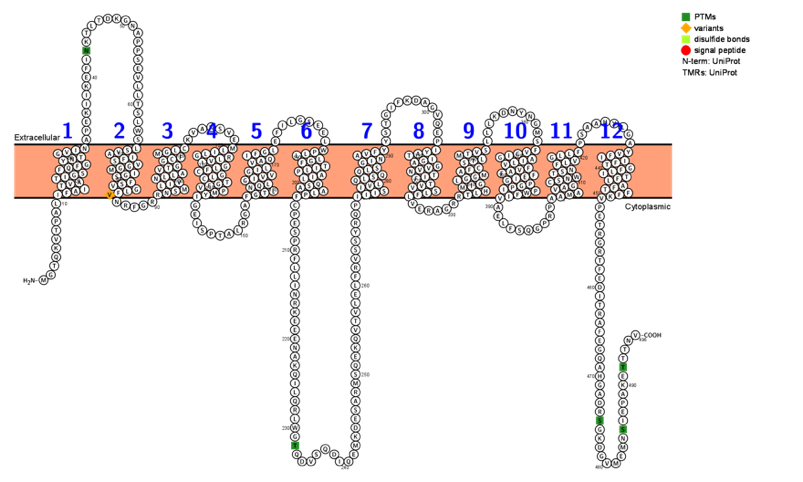

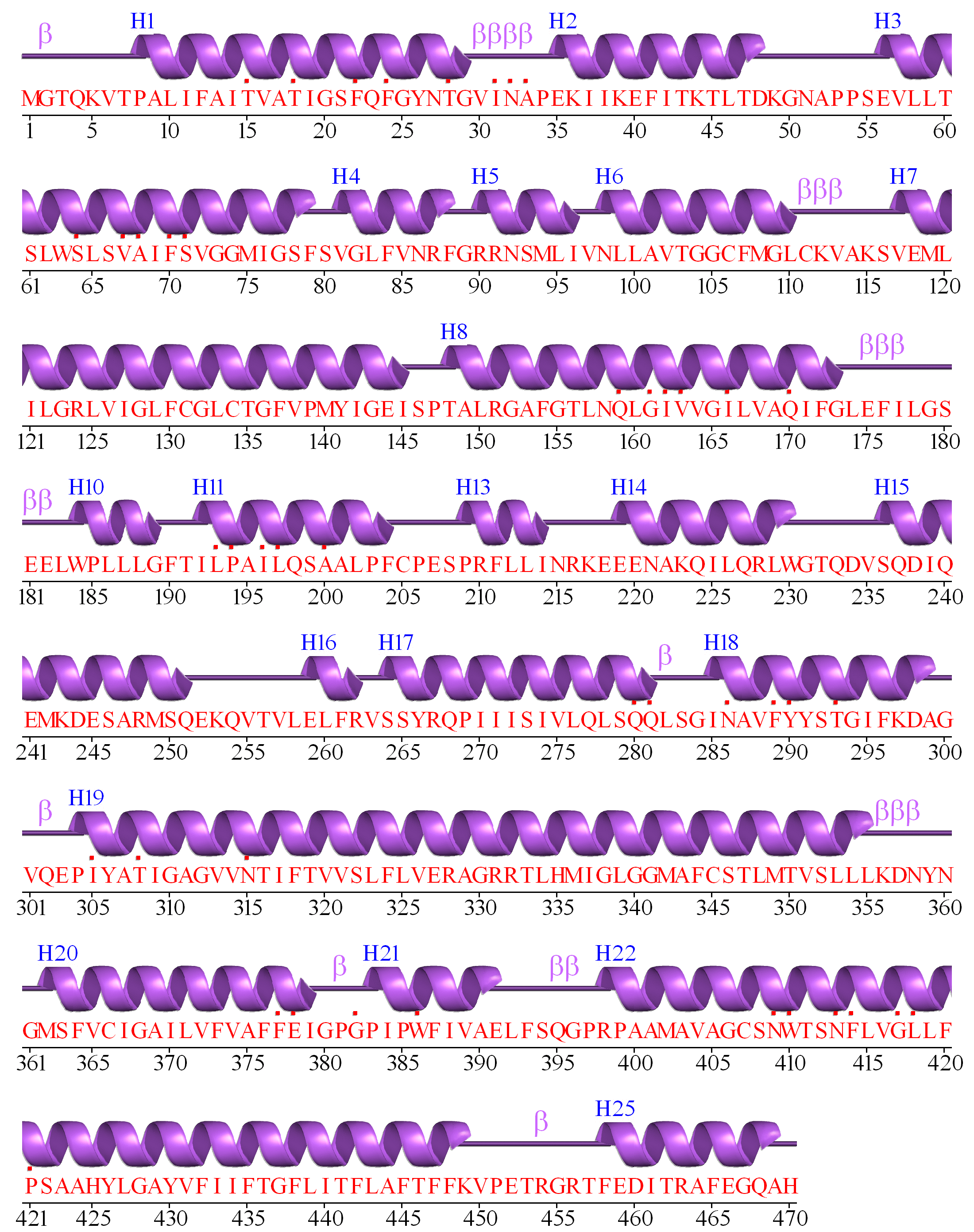


TM8-9

GTR3=GLUT3

[P11169](https://www.uniprot.org/uniprot/P11169)|GTR3 HUMAN Solute carrier family 2, facilitated glucose transporter member 3

MGTQKVTPALIFAITVATIGSFQFGYNTGVINAPEKIIKEFINKTLTDKGNAPPSEVLLT

SLWSLSVAIFSVGGMIGSFSVGLFVNRFGRRNSMLIVNLLAVTGGCFMGLCKVAKSVEML

ILGRLVIGLFCGLCTGFVPMYIGEISPTALRGAFGTLNQLGIVVGILVAQIFGLEFILGS

EELWPLLLGFTILPAILQSAALPFCPESPRFLLINRKEEENAKQILQRLWGTQDVSQDIQ

EMKDESARMSQEKQVTVLELFRVSSYRQPIIISIVLQLSQQLSGINAVFYYSTGIFKDAG

VQEPIYATIGAGVVNTIFTVVSLFLVERAGRRTLHMIGLGGMAFCSTLMTVSLLLKDNYN TM8-9

GMSFVCIGAILVFVAFFEIGPGPIPWFIVAELFSQGPRPAAMAVAGCSNWTSNFLVGLLF

PSAAHYLGAYVFIIFTGFLITFLAFTFFKVPETRGRTFEDITRAFEGQAHGADRSGKDGV

MEMNSIEPAKETTTNV


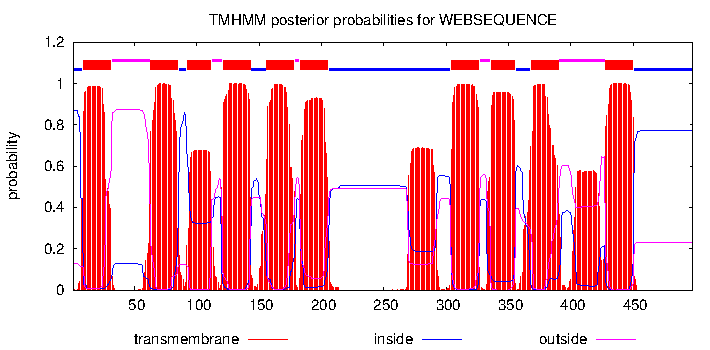

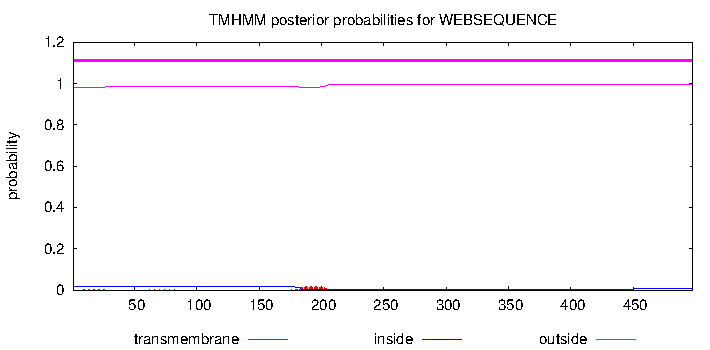


GLUT3^QTY^

MGTQKVTPALTYATTTATTGSYQYGYNTGTTNAPEKIIKEFINKTLTDKGNAPPSEVLLT

SLWSQSTATYSTGGMTGSYSTGQYTNRFGRRNSMQTTNQQATTGGCYMGQCKVAKSVEMQ

TQGRQTTGQYCGQCTGYTPMYTGEISPTALRGAYGTQNQQGTTTGTQTAQTYGQEFILGS

EELWPQQQGYTTQPATQQSAAQPYCPESPRFLLINRKEEENAKQILQRLWGTQDVSQDIQ

EMKDESARMSQEKQVTVLELFRVSSYRQPTTTSTTQQQSQQQSGTNATYYYSTGIFKDAG

VQEPTYATTGAGTTNTTYTTTSQYQTERAGRRTQHMTGQGGMAYCSTQMTTSQQQKDNYN TM8-9

GMSYTCTGATQTYTAYYETGPGPTPWYTTAELFSQGPRPAAMATAGCSNWTSNYQTGQQY

PSAAHYLGAYTYTTYTGYQTTYQAYTYYKTPETRGRTFEDITRAFEGQAHGADRSGKDGV

MEMNSIEPAKETTTNV

GTR4=GLUT4=SLC2A4= facilitated glucose transporter member 4, [P14672](https://www.uniprot.org/uniprot/P14672), 509aa


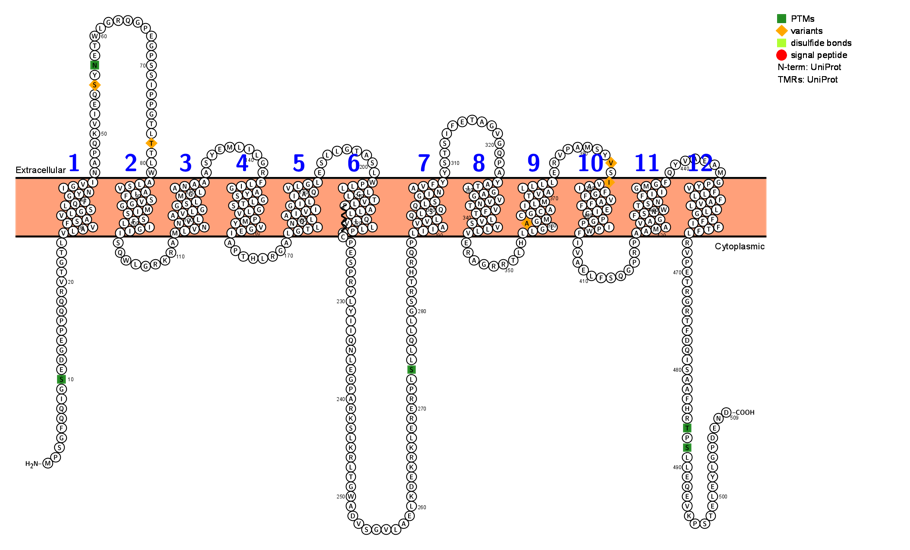


GTR4=GLUT4

P14672|GLUT4 HUMAN Solute carrier family 2, facilitated glucose transporter member 4

MPSGFQQIGSEDGEPPQQRVTGTLVLAVFSAVLGSLQFGYNIGVINAPQKVIEQSYNETW

LGRQGPEGPSSIPPGTLTTLWALSVAIFSVGGMISSFLIGIISQWLGRKRAMLVNNVLAV

LGGSLMGLANAAASYEMLILGRFLIGAYSGLTSGLVPMYVGEIAPTHLRGALGTLNQLAI

VIGILIAQVLGLESLLGTASLWPLLLGLTVLPALLQLVLLPFCPESPRYLYIIQNLEGPA

RKSLKRLTGWADVSGVLAELKDEKRKLERERPLSLLQLLGSRTHRQPLIIAVVLQLSQQL

SGINAVFYYSTSIFETAGVGQPAYATIGAGVVNTVFTLVSVLLVERAGRRTLHLLGLAGM

CGCAILMTVALLLLERVPAMSYVSIVAIFGFVAFFEIGPGPIPWFIVAELFSQGPRPAAM

AVAGFSNWTSNFIIGMGFQYVAEAMGPYVFLLFAVLLLGFFIFTFLRVPETRGRTFDQIS

AAFHRTPSLLEQEVKPSTELEYLGPDEND


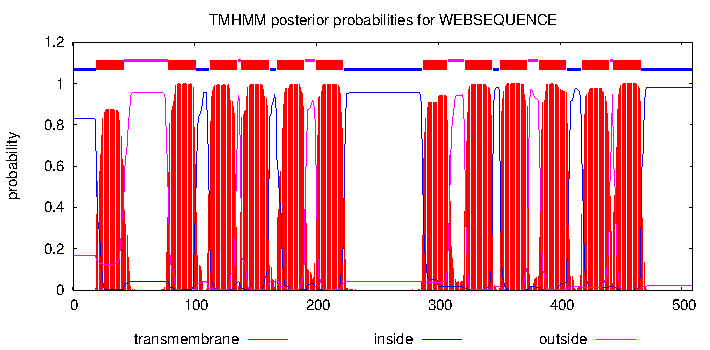

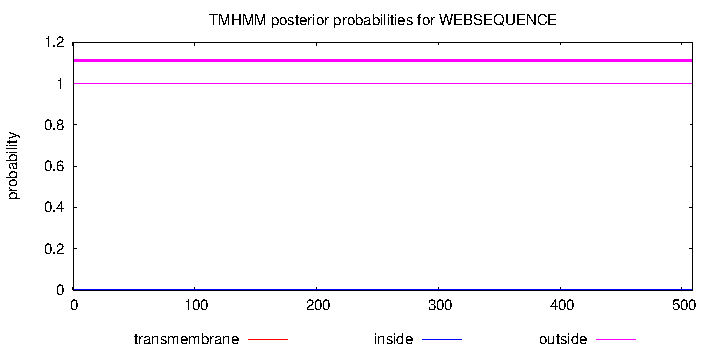


GLUT4^QTY^

MPSGFQQIGSEDGEPPQQRVTGTLTQATYSATQGSQQYGYNTGTTNAPQKVIEQSYNETW

LGRQGPEGPSSIPPGTLTTLWAQSTATYSTGGMTSSYQTGTTSQWLGRKRAMQTNNTQAT

QGGSQMGQANAAASYEMLILGRYQTGAYSGQTSGQTPMYTGETAPTHLRGAQGTQNQQAT

TTGTQTAQTQGQESLLGTASLWPQQQGQTTQPAQQQQTQQPYCPESPRYLYIIQNLEGPA

RKSLKRLTGWADVSGVLAELKDEKRKLERERPLSLLQLLGSRTHRQPQTTATTQQQSQQQ

SGTNATYYYSTSIFETAGVGQPAYATTGAGTTNTTYTQTSTQQTERAGRRTLHQQGQAGM

CGCATQMTTAQQQQERVPAMSYVSTTATYGYTAYYETGPGPTPWYIVAELFSQGPRPAAM

ATAGYSNWTSNYTTGMGYQYVAEAMGPYTYQQYATQQQGYYTYTYQRVPETRGRTFDQIS

AAFHRTPSLLEQEVKPSTELEYLGPDEND

GTR5=GLUT5=SLC2A5= facilitated glucose transporter member 5, [P22732](https://www.uniprot.org/uniprot/P22732), 501aa


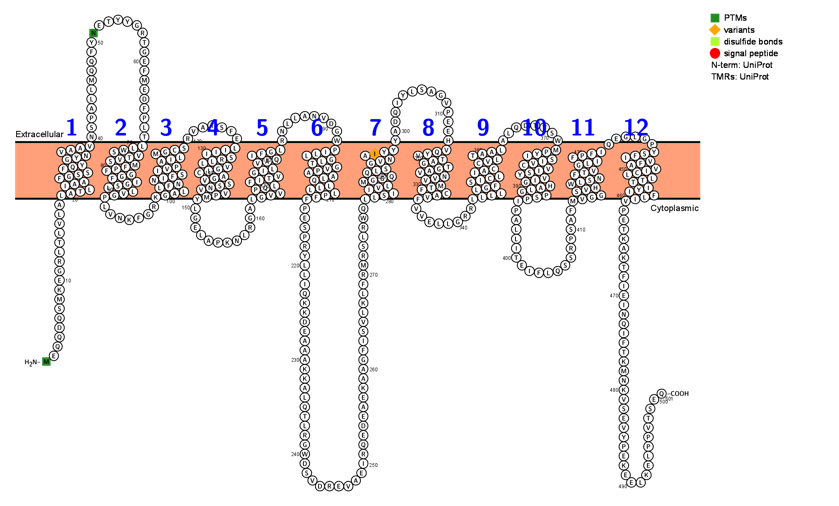


[P22732](https://www.uniprot.org/uniprot/P22732)| GTR5=GLUT5 Solute carrier family 2, facilitated glucose transporter member 5

MEQQDQSMKEGRLTLVLALATLIAAFGSSFQYGYNVAAVNSPALLMQQFYNETYYGRTGE

FMEDFPLTLLWSVTVSMFPFGGFIGSLLVGPLVNKFGRKGALLFNNIFSIVPAILMGCSR

VATSFELIIISRLLVGICAGVSSNVVPMYLGELAPKNLRGALGVVPQLFITVGILVAQIF

GLRNLLANVDGWPILLGLTGVPAALQLLLLPFFPESPRYLLIQKKDEAAAKKALQTLRGW

DSVDREVAEIRQEDEAEKAAGFISVLKLFRMRSLRWQLLSIIVLMGGQQLSGVNAIYYYA

DQIYLSAGVPEEHVQYVTAGTGAVNVVMTFCAVFVVELLGRRLLLLLGFSICLIACCVLT

AALALQDTVSWMPYISIVCVISYVIGHALGPSPIPALLITEIFLQSSRPSAFMVGGSVHW

LSNFTVGLIFPFIQEGLGPYSFIVFAVICLLTTIYIFLIVPETKAKTFIEINQIFTKMNK

VSEVYPEKEELKELPPVTSEQ


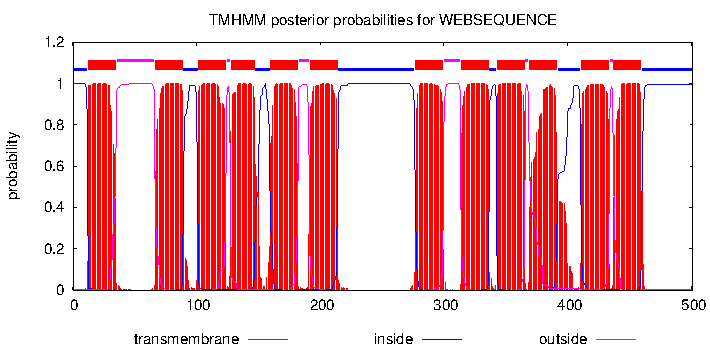

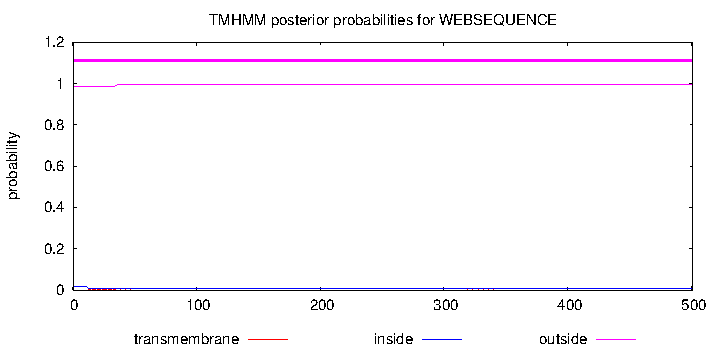


GLUT5^QTY^

MEQQDQSMKEGRLTLVLAQATQTAAYGSSYQYGYNTAATNSPALLMQQFYNETYYGRTGE

FMEDFPLTQQWSTTTSMYPYGGYTGSQQTGPLVNKFGRKGAQQYNNTYSTTPATQMGCSR

VATSFEQTTTSRQQTGTCAGTSSNTTPMYLGELAPKNLRGAQGTTPQQYTTTGTQTAQTY

GQRNLLANVDGWPTQQGQTGTPAAQQQQQQPYYPESPRYLLIQKKDEAAAKKALQTLRGW

DSVDREVAEIRQEDEAEKAAGFISVLKLFRMRSLRWQQQSTTTQMGGQQQSGTNATYYYA

DQIYLSAGVPEEHTQYTTAGTGATNTTMTYCATYVVELLGRRQQQQQGFSTCQTACCTQT

AAQALQDTVSWMPYTSTTCTTSYTTGHAQGPSPTPALLITEIFLQSSRPSAFMTGGSTHW

QSNYTTGQTYPYTQEGLGPYSYTTYATTCQQTTTYTYQTTPETKAKTFIEINQIFTKMNK

VSEVYPEKEELKELPPVTSEQ

GTR6=GLUT6=SLC2A6= facilitated glucose transporter member 6, [Q9UGQ3](https://www.uniprot.org/uniprot/Q9UGQ3), 507aa


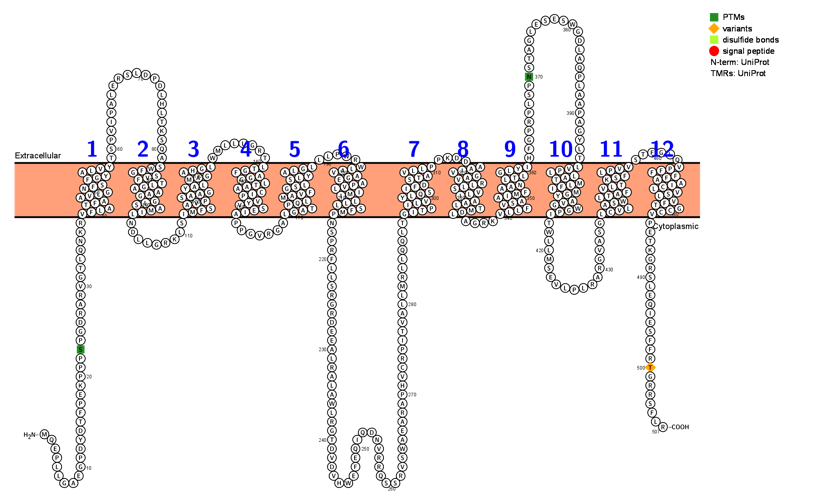


[Q9UGQ3](https://www.uniprot.org/uniprot/Q9UGQ3)| GTR6=GLUT6 Solute carrier family 2, facilitated glucose transporter member 6

MQEPLLGAEGPDYDTFPEKPPPSPGDRARVGTLQNKRVFLATFAAVLGNFSFGYALVYTS

PVIPALERSLDPDLHLTKSQASWFGSVFTLGAAAGGLSAMILNDLLGRKLSIMFSAVPSA

AGYALMAGAHGLWMLLLGRTLTGFAGGLTAACIPVYVSEIAPPGVRGALGATPQLMAVFG

SLSLYALGLLLPWRWLAVAGEAPVLIMILLLSFMPNSPRFLLSRGRDEEALRALAWLRGT

DVDVHWEFEQIQDNVRRQSSRVSWAEARAPHVCRPITVALLMRLLQQLTGITPILVYLQS

IFDSTAVLLPPKDDAAIVGAVRLLSVLIAALTMDLAGRKVLLFVSAAIMFAANLTLGLYI

HFGPRPLSPNSTAGLESESWGDLAQPLAAPAGYLTLVPLLATMLFIMGYAVGWGPITWLL

MSEVLPLRARGVASGLCVLASWLTAFVLTKSFLPVVSTFGLQVPFFFFAAICLVSLVFTG

CCVPETKGRSLEQIESFFRTGRRSFLR


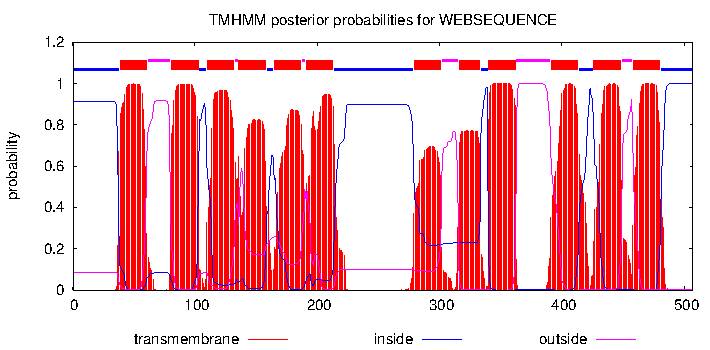

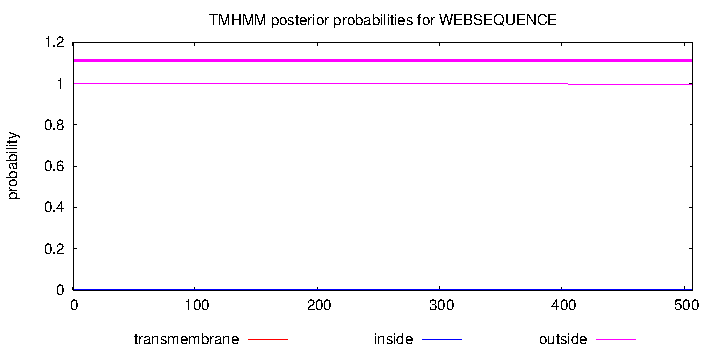


GLUT6^QTY^

MQEPLLGAEGPDYDTFPEKPPPSPGDRARVGTLQNKRTYQATYAATQGNYSYGYAQTYTS

PVIPALERSLDPDLHLTKSQASWYGSTYTQGAAAGGQSAMTQNDLLGRKLSTMYSATPSA

AGYAQMAGAHGQWMLLLGRTQTGYAGGQTAACTPTYTSETAPPGVRGAQGATPQQMATYG

SQSQYAQGQLLPWRWQATAGEAPTQTMTQQQSYMPNSPRFLLSRGRDEEALRALAWLRGT

DVDVHWEFEQIQDNVRRQSSRVSWAEARAPHVCRPITVALLMRLLQQLTGTTPTQTYQQS

TYDSTATQQPPKDDAATTGATRQQSTQTAAQTMDQAGRKTQQYTSAATMYAANQTQGQYT

HFGPRPLSPNSTAGLESESWGDLAQPLAAPAGYLTQTPQQATMQYTMGYATGWGPTTWLL

MSEVLPLRARGVASGQCTQASWQTAYTQTKSYQPTTSTFGLQVPYYYYAATCQTSQTYTG

CCTPETKGRSLEQIESFFRTGRRSFLR

GTR7=GLUT7=SLC2A7= facilitated glucose transporter member 7, [Q6PXP3](https://www.uniprot.org/uniprot/Q6PXP3), 512aa


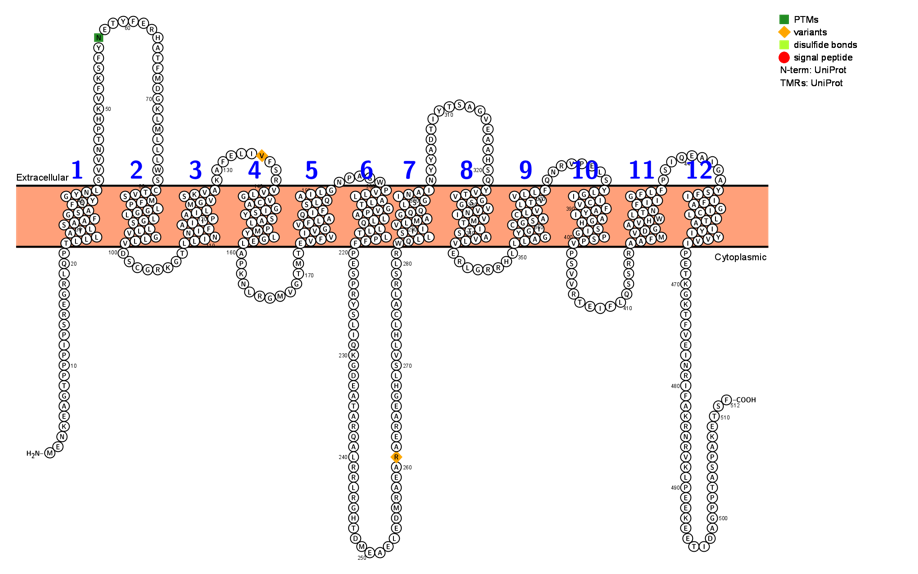


[Q6PXP3](https://www.uniprot.org/uniprot/Q6PXP3)| GTR7=GLUT7 Solute carrier family 2, facilitated glucose transporter member 7

MENKEAGTPPPIPSREGRLQPTLLLATLSAAFGSAFQYGYNLSVVNTPHKVFKSFYNETY

FERHATFMDGKLMLLLWSCTVSMFPLGGLLGSLLVGLLVDSCGRKGTLLINNIFAIIPAI

LMGVSKVAKAFELIVFSRVVLGVCAGISYSALPMYLGELAPKNLRGMVGTMTEVFVIVGV

FLAQIFSLQAILGNPAGWPVLLALTGVPALLQLLTLPFFPESPRYSLIQKGDEATARQAL

RRLRGHTDMEAELEDMRAEARAERAEGHLSVLHLCALRSLRWQLLSIIVLMAGQQLSGIN

AINYYADTIYTSAGVEAAHSQYVTVGSGVVNIVMTITSAVLVERLGRRHLLLAGYGICGS

ACLVLTVVLLFQNRVPELSYLGIICVFAYIAGHSIGPSPVPSVVRTEIFLQSSRRAAFMV

DGAVHWLTNFIIGFLFPSIQEAIGAYSFIIFAGICLLTAIYIYVVIPETKGKTFVEINRI

FAKRNRVKLPEEKEETIDAGPPTASPAKETSF


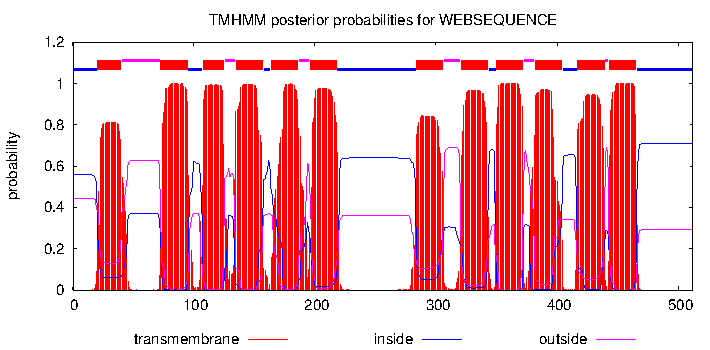

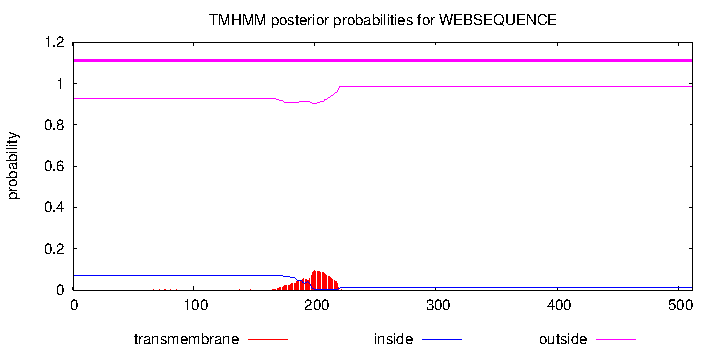


GLUT7^QTY^

MENKEAGTPPPIPSREGRLQPTQQQATQSAAYGSAYQYGYNQSVVNTPHKVFKSFYNETY

FERHATFMDGKLMLLLWSCTTSMYPQGGQQGSQQTGQQTDSCGRKGTQQTNNTYATTPAT

QMGTSKTAKAFELIVFSRTTQGTCAGTSYSAQPMYQGEQAPKNLRGMVGTMTETYTTTGT

YQAQTYSQQATQGNPAGWPTQQAQTGTPAQQQQQTQPYYPESPRYSLIQKGDEATARQAL

RRLRGHTDMEAELEDMRAEARAERAEGHLSVLHLCALRSLRWQQQSTTTQMAGQQQSGTN

ATNYYADTIYTSAGVEAAHSQYTTTGSGTTNTTMTTTSATQTERLGRRHLQQAGYGTCGS

ACQTQTTTQQYQNRVPELSYQGTTCTYAYTAGHSTGPSPTPSVVRTEIFLQSSRRAAYMT

DGATHWQTNYTTGYQYPSIQEAIGAYSYTTYAGTCQQTATYTYTTTPETKGKTFVEINRI

FAKRNRVKLPEEKEETIDAGPPTASPAKETSF

GTR8=GLUT8= SLC2A8= facilitated glucose transporter member 8, [Q9NY64](https://www.uniprot.org/uniprot/Q9NY64), 477aa


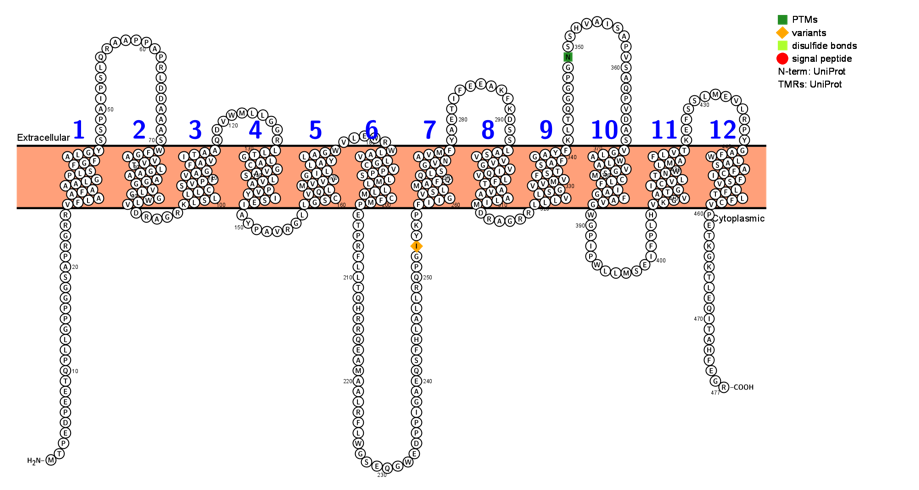


Q9NY64|GTR8=GLUT8 Solute carrier family 2, facilitated glucose transporter member 8, 477aa

MTPEDPEETQPLLGPPGGSAPRGRRVFLAAFAAALGPLSFGFALGYSSPAIPSLQRAAPP

APRLDDAAASWFGAVVTLGAAAGGVLGGWLVDRAGRKLSLLLCSVPFVAGFAVITAAQDV

WMLLGGRLLTGLACGVASLVAPVYISEIAYPAVRGLLGSCVQLMVVVGILLAYLAGWVLE

WRWLAVLGCVPPSLMLLLMCFMPETPRFLLTQHRRQEAMAALRFLWGSEQGWEDPPIGAE

QSFHLALLRQPGIYKPFIIGVSLMAFQQLSGVNAVMFYAETIFEEAKFKDSSLASVVVGV

IQVLFTAVAALIMDRAGRRLLLVLSGVVMVFSTSAFGAYFKLTQGGPGNSSHVAISAPVS

AQPVDASVGLAWLAVGSMCLFIAGFAVGWGPIPWLLMSEIFPLHVKGVATGICVLTNWLM

AFLVTKEFSSLMEVLRPYGAFWLASAFCIFSVLFTLFCVPETKGKTLEQITAHFEGR


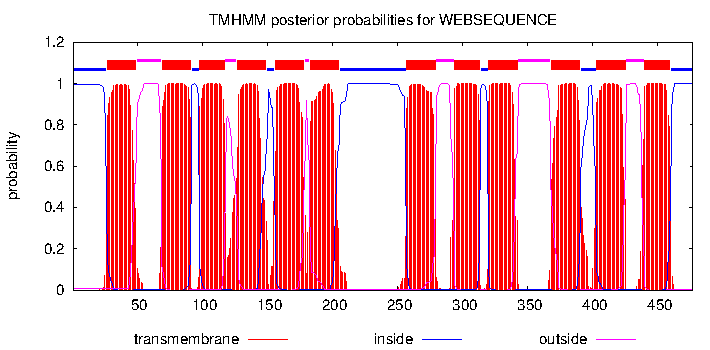

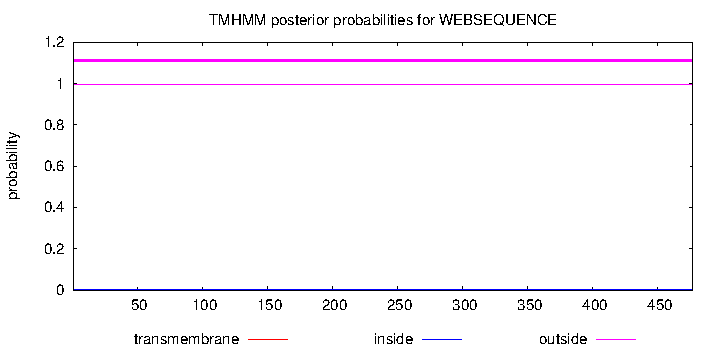


GLUT8^QTY^

MTPEDPEETQPLLGPPGGSAPRGRRTYQAAYAAAQGPQSYGYAQGYSSPAIPSLQRAAPP

APRLDDAAASWYGATTTQGAAAGGTQGGWQTDRAGRKQSQQQCSTPYTAGYATTTAAQDV

WMLLGGRQQTGQACGTASQTAPTYTSETAYPAVRGLQGSCTQQMTTTGTQQAYQAGWTLE

WRWQATQGCTPPSQMQQQMCYMPETPRFLLTQHRRQEAMAALRFLWGSEQGWEDPPIGAE

QSFHLALLRQPGIYKPYTTGTSQMAYQQQSGTNATMYYAETIFEEAKFKDSSQASTTTGT

TQTQYTATAAQTMDRAGRRQQQTQSGTTMTYSTSAYGAYYKLTQGGPGNSSHVAISAPVS

AQPVDASTGQAWQATGSMCQYTAGYATGWGPIPWLLMSEIFPLHTKGTATGTCTQTNWQM

AYQTTKEFSSLMEVLRPYGAYWQASAYCTYSTQYTQYCTPETKGKTLEQITAHFEGR

GTR9=GLUT9=SLC2A9= facilitated glucose transporter member 9, [Q9NRM0](https://www.uniprot.org/uniprot/Q9NRM0), 540aa


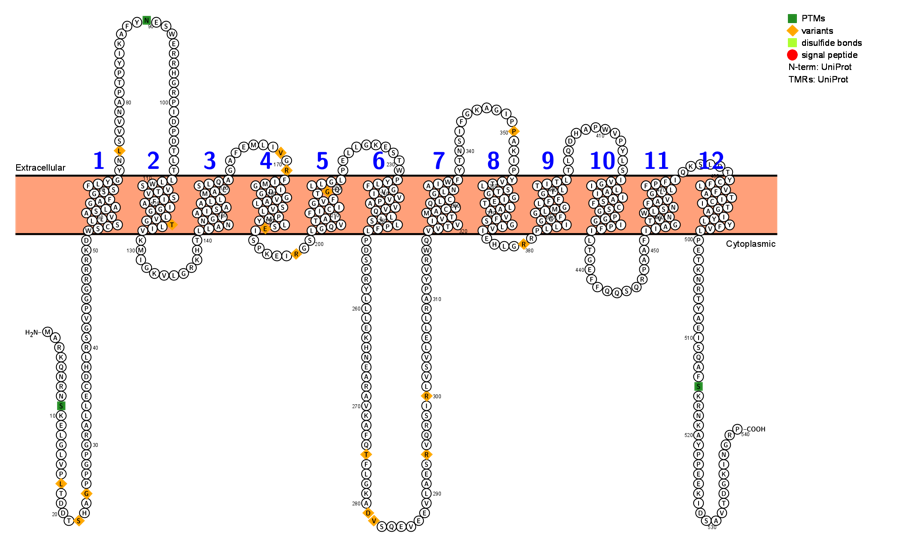


[Q9NRM0](https://www.uniprot.org/uniprot/Q9NRM0) | GTR9=GLUT9 Solute carrier family 2, facilitated glucose transporter member 9

MARKQNRNSKELGLVPLTDDTSHAGPPGPGRALLECDHLRSGVPGGRRRKDWSCSLLVAS

LAGAFGSSFLYGYNLSVVNAPTPYIKAFYNESWERRHGRPIDPDTLTLLWSVTVSIFAIG

GLVGTLIVKMIGKVLGRKHTLLANNGFAISAALLMACSLQAGAFEMLIVGRFIMGIDGGV

ALSVLPMYLSEISPKEIRGSLGQVTAIFICIGVFTGQLLGLPELLGKESTWPYLFGVIVV

PAVVQLLSLPFLPDSPRYLLLEKHNEARAVKAFQTFLGKADVSQEVEEVLAESRVQRSIR

LVSVLELLRAPYVRWQVVTVIVTMACYQLCGLNAIWFYTNSIFGKAGIPPAKIPYVTLST

GGIETLAAVFSGLVIEHLGRRPLLIGGFGLMGLFFGTLTITLTLQDHAPWVPYLSIVGIL

AIIASFCSGPGGIPFILTGEFFQQSQRPAAFIIAGTVNWLSNFAVGLLFPFIQKSLDTYC

FLVFATICITGAIYLYFVLPETKNRTYAEISQAFSKRNKAYPPEEKIDSAVTDGKINGRP


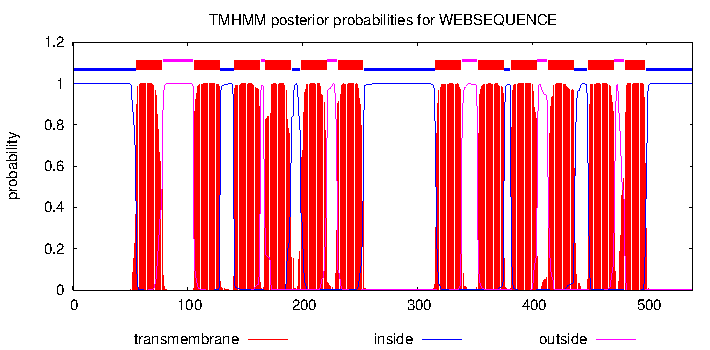

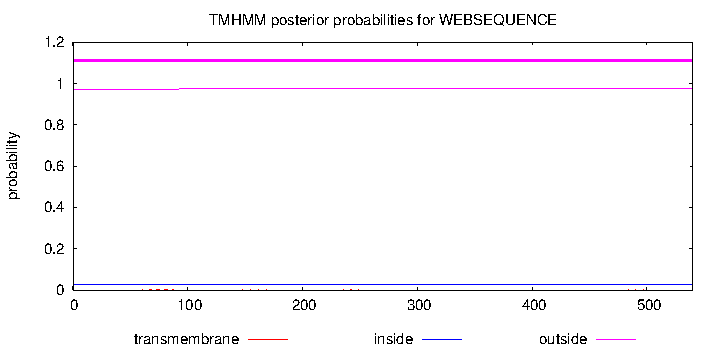


GLUT9^QTY^

MARKQNRNSKELGLVPLTDDTSHAGPPGPGRALLECDHLRSGVPGGRRRKDWSCSQQTAS

QAGAYGSSYQYGYNLSVVNAPTPYIKAFYNESWERRHGRPIDPDTLTQQWSTTTSTYATG

GQTGTQTTKMIGKVLGRKHTQQANNGYATSAAQQMACSQQAGAFEMLIVGRYTMGTDGGT

AQSTQPMYQSETSPKEIRGSQGQTTATYTCTGTYTGQQQGQPELLGKESTWPYQYGTTTT

PATTQQQSQPYQPDSPRYLLLEKHNEARAVKAFQTFLGKADVSQEVEEVLAESRVQRSIR

LVSVLELLRAPYVRWQTTTTTTTMACYQQCGQNATWYYTNSIFGKAGIPPAKIPYTTQST

GGTETQAATYSGQTTEHLGRRPQQTGGYGQMGQYYGTQTTTQTLQDHAPWVPYLSTTGTQ

ATTASYCSGPGGTPYTQTGEFFQQSQRPAAFTTAGTTNWQSNYATGQQYPYTQKSLDTYC

YQTYATTCTTGATYQYYTQPETKNRTYAEISQAFSKRNKAYPPEEKIDSAVTDGKINGRP

GTR10=GLUT10=SLC2A10= facilitated glucose transporter member 10, [O95528](https://www.uniprot.org/uniprot/O95528), 541aa


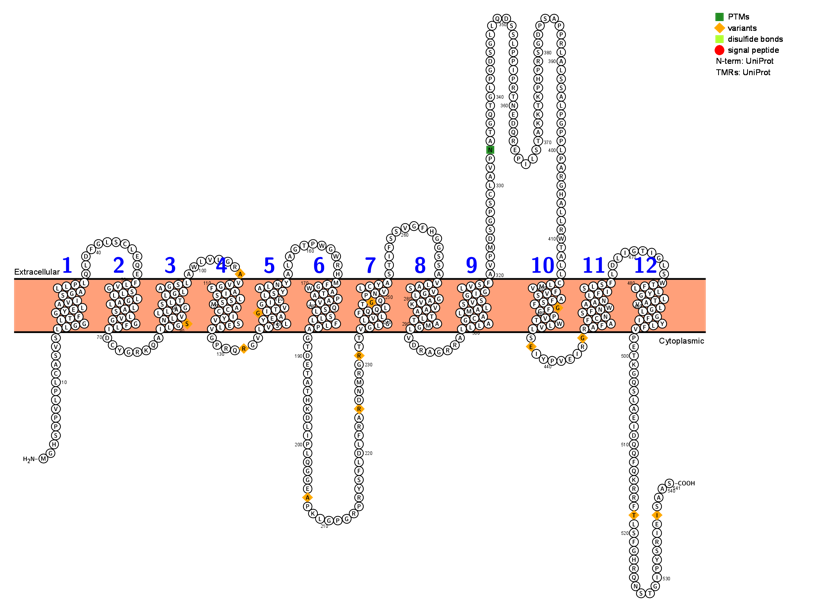


[O95528](https://www.uniprot.org/uniprot/O95528)| GTR10=GLUT10 Solute carrier family 2, facilitated glucose transporter member 10

MGHSPPVLPLCASVSLLGGLTFGYELAVISGALLPLQLDFGLSCLEQEFLVGSLLLGALL

ASLVGGFLIDCYGRKQAILGSNLVLLAGSLTLGLAGSLAWLVLGRAVVGFAISLSSMACC

IYVSELVGPRQRGVLVSLYEAGITVGILLSYALNYALAGTPWGWRHMFGWATAPAVLQSL

SLLFLPAGTDETATHKDLIPLQGGEAPKLGPGRPRYSFLDLFRARDNMRGRTTVGLGLVL

FQQLTGQPNVLCYASTIFSSVGFHGGSSAVLASVGLGAVKVAATLTAMGLVDRAGRRALL

LAGCALMALSVSGIGLVSFAVPMDSGPSCLAVPNATGQTGLPGDSGLLQDSSLPPIPRTN

EDQREPILSTAKKTKPHPRSGDPSAPPRLALSSALPGPPLPARGHALLRWTALLCLMVFV

SAFSFGFGPVTWLVLSEIYPVEIRGRAFAFCNSFNWAANLFISLSFLDLIGTIGLSWTFL

LYGLTAVLGLGFIYLFVPETKGQSLAEIDQQFQKRRFTLSFGHRQNSTGIPYSRIEISAAS


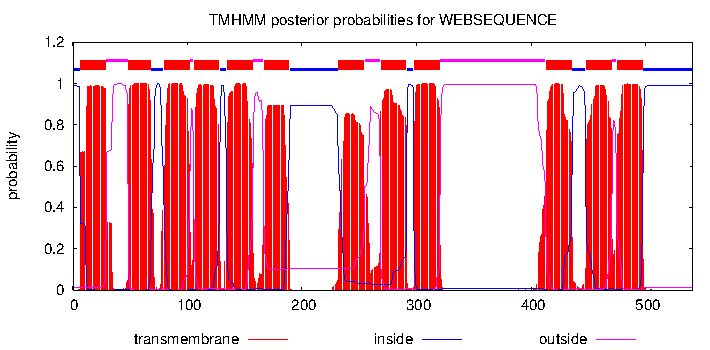

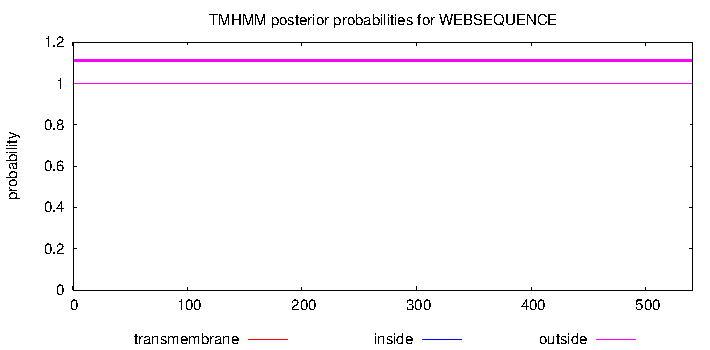


GLUT10^QTY^

MGHSPPVLPLCASVSQQGGQTYGYEQATTSGAQQPQQLDFGLSCLEQEYQTGSQQQGAQQ

ASQTGGYQTDCYGRKQATQGSNQTQQAGSQTQGQAGSQAWLVLGRATTGYATSQSSMACC

TYTSEQTGPRQRGVQTSQYEAGTTTGTQQSYAQNYALAGTPWGWRHMYGWATAPATQQSQ

SQQYQPAGTDETATHKDLIPLQGGEAPKLGPGRPRYSFLDLFRARDNMRGRTTTGQGQTQ

YQQQTGQPNTQCYASTIFSSVGFHGGSSATQASTGQGATKTAATQTAMGQVDRAGRRAQQ

QAGCAQMAQSVSGTGQTSYAVPMDSGPSCLAVPNATGQTGLPGDSGLLQDSSLPPIPRTN

EDQREPILSTAKKTKPHPRSGDPSAPPRLALSSALPGPPLPARGHALLRWTAQQCQMTYT

SAYSYGYGPTTWQTQSEIYPVEIRGRAYAYCNSYNWAANQYTSQSYQDLIGTIGLSWTYQ

QYGQTATQGQGYTYQYTPETKGQSLAEIDQQFQKRRFTLSFGHRQNSTGIPYSRIEISAAS

GTR11=GLUT11=SLC2A11=, facilitated glucose transporter member 11, [Q9BYW1](https://www.uniprot.org/uniprot/Q9BYW1), 496aa


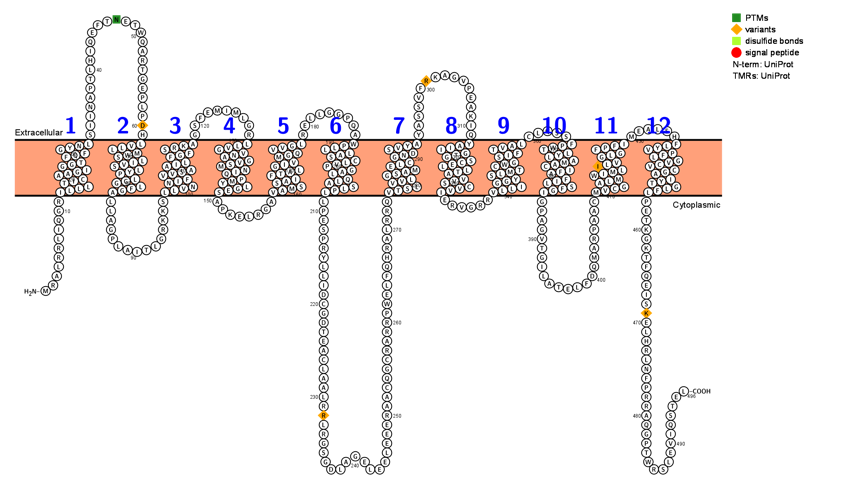


[Q9BYW1](https://www.uniprot.org/uniprot/Q9BYW1)| GTR11=GLUT11 Solute carrier family 2, facilitated glucose transporter member 11

MRALRRLIQGRILLLTICAAGIGGTFQFGYNLSIINAPTLHIQEFTNETWQARTGEPLPD

HLVLLMWSLIVSLYPLGGLFGALLAGPLAITLGRKKSLLVNNIFVVSAAILFGFSRKAGS

FEMIMLGRLLVGVNAGVSMNIQPMYLGESAPKELRGAVAMSSAIFTALGIVMGQVVGLRE

LLGGPQAWPLLLASCLVPGALQLASLPLLPESPRYLLIDCGDTEACLAALRRLRGSGDLA

GELEELEEERAACQGCRARRPWELFQHRALRRQVTSLVVLGSAMELCGNDSVYAYASSVF

RKAGVPEAKIQYAIIGTGSCELLTAVVSCVVIERVGRRVLLIGGYSLMTCWGSIFTVALC

LQSSFPWTLYLAMACIFAFILSFGIGPAGVTGILATELFDQMARPAACMVCGALMWIMLI

LVGLGFPFIMEALSHFLYVPFLGVCVCGAIYTGLFLPETKGKTFQEISKELHRLNFPRRA

QGPTWRSLEVIQSTEL


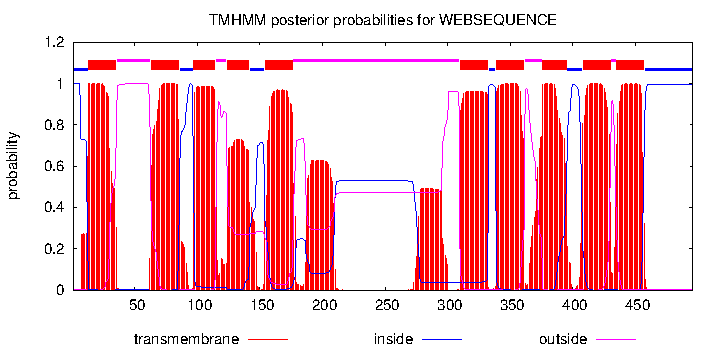

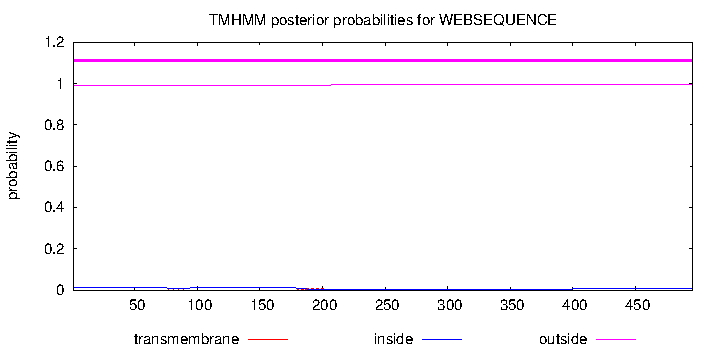


GLUT11^QTY^

MRALRRLIQGRTQQQTTCAAGTGGTYQYGYNQSIINAPTLHIQEFTNETWQARTGEPLPD

HQTQQMWSQTTSQYPQGGQYGALLAGPLAITLGRKKSQQTNNTYTTSAATQYGYSRKAGS

FEMIMLGRQQTGTNAGTSMNTQPMYQGESAPKELRGATAMSSATYTAQGTTMGQTTGQRE

LLGGPQAWPLLLASCLVPGALQLASLPLLPESPRYLLIDCGDTEACLAALRRLRGSGDLA

GELEELEEERAACQGCRARRPWELFQHRALRRQTTSQTTQGSAMEQCGNDSTYAYASSVF

RKAGVPEAKIQYATTGTGSCEQQTATTSCTTTERVGRRTQQTGGYSQMTCWGSTYTTAQC

LQSSYPWTQYQAMACTYAYTQSYGTGPAGVTGILATELFDQMARPAACMTCGAQMWTMQT

QTGQGYPYTMEALSHYQYTPYQGTCTCGATYTGQYQPETKGKTFQEISKELHRLNFPRRA

QGPTWRSLEVIQSTEL

GLUT12=GTR12=SLC2A12= facilitated glucose transporter member 12, [Q8TD20](https://www.uniprot.org/uniprot/Q8TD20), 617aa


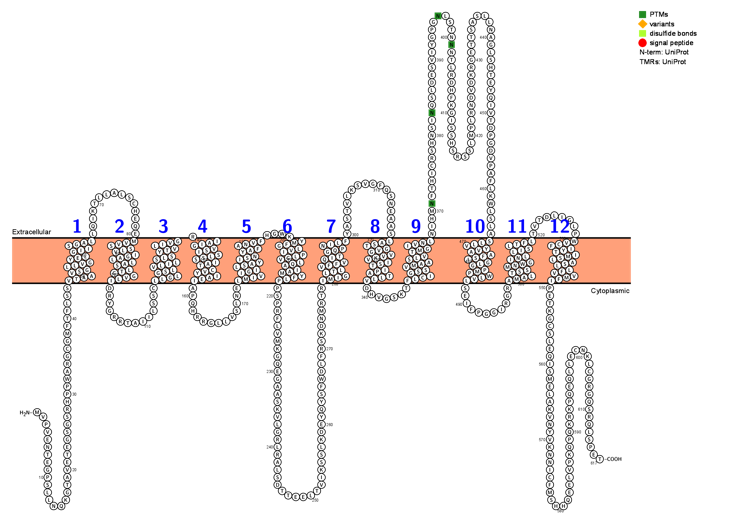


[Q8TD20](https://www.uniprot.org/uniprot/Q8TD20)| GLUT12=GTR12 Solute carrier family 2, facilitated glucose transporter member 12

MVPVENTEGPSLLNQKGTAVETEGSGSRHPPWARGCGMFTFLSSVTAAVSGLLVGYELGI

ISGALLQIKTLLALSCHEQEMVVSSLVIGALLASLTGGVLIDRYGRRTAIILSSCLLGLG

SLVLILSLSYTVLIVGRIAIGVSISLSSIATCVYIAEIAPQHRRGLLVSLNELMIVIGIL

SAYISNYAFANVFHGWKYMFGLVIPLGVLQAIAMYFLPPSPRFLVMKGQEGAASKVLGRL

RALSDTTEELTVIKSSLKDEYQYSFWDLFRSKDNMRTRIMIGLTLVFFVQITGQPNILFY

ASTVLKSVGFQSNEAASLASTGVGVVKVISTIPATLLVDHVGSKTFLCIGSSVMAASLVT

MGIVNLNIHMNFTHICRSHNSINQSLDESVIYGPGNLSTNNNTLRDHFKGISSHSRSSLM

PLRNDVDKRGETTSASLLNAGLSHTEYQIVTDPGDVPAFLKWLSLASLLVYVAAFSIGLG

PMPWLVLSEIFPGGIRGRAMALTSSMNWGINLLISLTFLTVTDLIGLPWVCFIYTIMSLA

SLLFVVMFIPETKGCSLEQISMELAKVNYVKNNICFMSHHQEELVPKQPQKRKPQEQLLE

CNKLCGRGQSRQLSPET


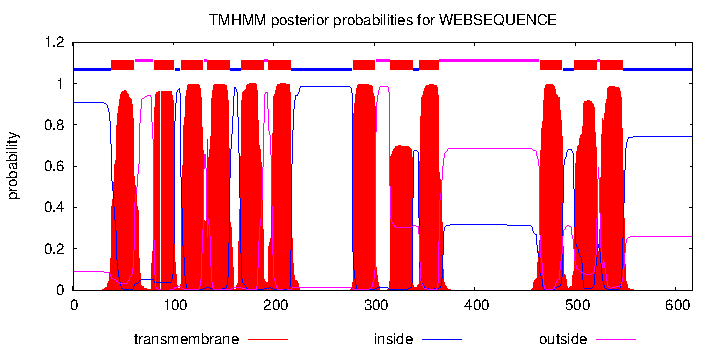

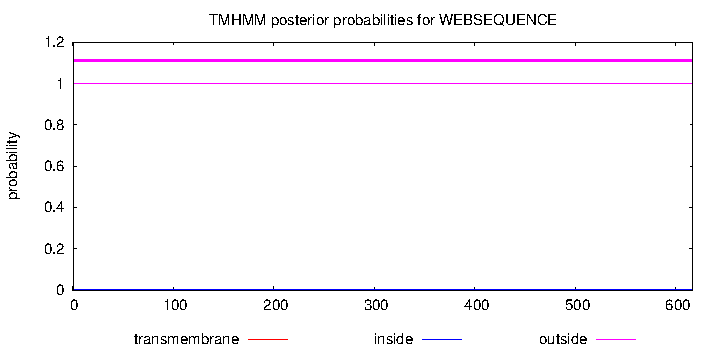


GLUT12^QTY^

MVPVENTEGPSLLNQKGTAVETEGSGSRHPPWARGCGMFTFLSSTTAATSGQQTGYEQGT

TSGAQQQIKTLLALSCHEQEMTTSSQTTGAQQASQTGGTQTDRYGRRTAIILSSCQQGQG

SQTQTQSQSYTTQTTGRTATGTSTSQSSTATCTYTAEIAPQHRRGLLVSLNEQMTTTGTQ

SAYTSNYAYANTYHGWKYMYGQTTPQGTQQATAMYYQPPSPRFLVMKGQEGAASKVLGRL

RALSDTTEELTVIKSSLKDEYQYSFWDLFRSKDNMRTRTMTGQTQTYYTQTTGQPNTQYY

ASTVLKSVGFQSNEAASQASTGTGTTKTTSTTPATQQTDHVGSKTYQCTGSSTMAASQTT

MGTTNQNIHMNFTHICRSHNSINQSLDESVIYGPGNLSTNNNTLRDHFKGISSHSRSSLM

PLRNDVDKRGETTSASLLNAGLSHTEYQIVTDPGDVPAFLKWLSLASQQTYTAAYSTGQG

PMPWQTQSEIFPGGIRGRAMAQTSSMNWGTNQQTSQTYQTVTDLIGLPWTCYTYTTMSQA

SQQYTTMYTPETKGCSLEQISMELAKVNYVKNNICFMSHHQEELVPKQPQKRKPQEQLLE

CNKLCGRGQSRQLSPET

GLUT13=SLC2A13=MYCT=HMIT, [Q96QE2](https://www.uniprot.org/uniprot/Q96QE2), 648aa


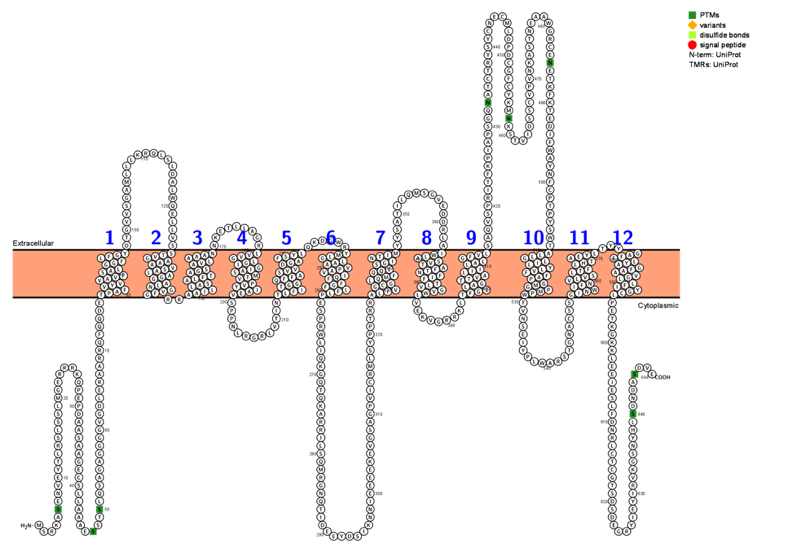


>sp|[Q96QE2](https://www.uniprot.org/uniprot/Q96QE2)|MYCT=GLUT13=SLC2A13 Proton myo-inositol cotransporter

MSRKASENVEYTLRSLSSLMGERRRKQPEPDAASAAGECSLLAAAESSTSLQSAGAGGGG

VGDLERAARRQFQQDETPAFVYVVAVFSALGGFLFGYDTGVVSGAMLLLKRQLSLDALWQ

ELLVSSTVGAAAVSALAGGALNGVFGRRAAILLASALFTAGSAVLAAANNKETLLAGRLV

VGLGIGIASMTVPVYIAEVSPPNLRGRLVTINTLFITGGQFFASVVDGAFSYLQKDGWRY

MLGLAAVPAVIQFFGFLFLPESPRWLIQKGQTQKARRILSQMRGNQTIDEEYDSIKNNIE

EEEKEVGSAGPVICRMLSYPPTRRALIVGCGLQMFQQLSGINTIMYYSATILQMSGVEDD

RLAIWLASVTAFTNFIFTLVGVWLVEKVGRRKLTFGSLAGTTVALIILALGFVLSAQVSP

RITFKPIAPSGQNATCTRYSYCNECMLDPDCGFCYKMNKSTVIDSSCVPVNKASTNEAAW

GRCENETKFKTEDIFWAYNFCPTPYSWTALLGLILYLVFFAPGMGPMPWTVNSEIYPLWA

RSTGNACSSGINWIFNVLVSLTFLHTAEYLTYYGAFFLYAGFAAVGLLFIYGCLPETKGK

KLEEIESLFDNRLCTCGTSDSDEGRYIEYIRVKGSNYHLSDNDASDVE


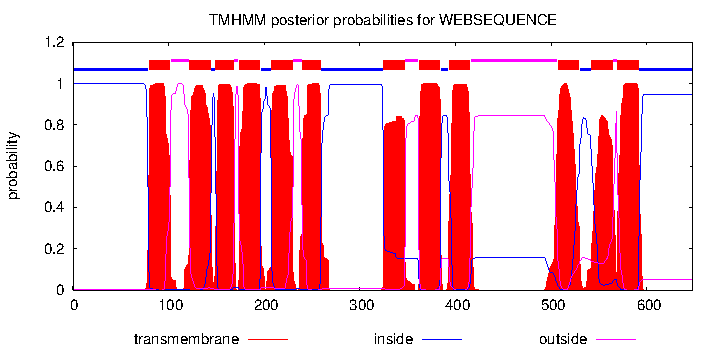

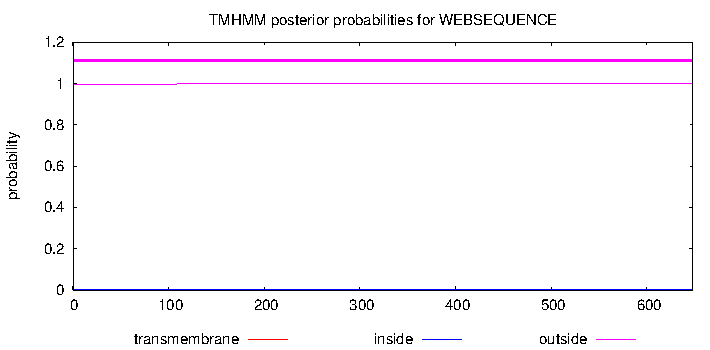


GLUT13^QTY^

MSRKASENVEYTLRSLSSLMGERRRKQPEPDAASAAGECSLLAAAESSTSLQSAGAGGGG

VGDLERAARRQFQQDETPAYTYTTATYSAQGGYQYGYDTGVVSGAMLLLKRQLSLDALWQ

ELLVSSTTGAAATSAQAGGAQNGTYGRRAATQQASAQYTAGSATQAAANNKETLLAGRQT

TGQGTGTASMTTPTYTAETSPPNLRGRLVTINTQYTTGGQYYASTTDGAYSYQQKDGWRY

MQGQAATPATTQYYGYQYQPESPRWLIQKGQTQKARRILSQMRGNQTIDEEYDSIKNNIE

EEEKEVGSAGPVICRMLSYPPTRRAQTTGCGQQMYQQQSGTNTTMYYSATILQMSGVEDD

RLATWQASTTAYTNYTYTQTGTWQVEKVGRRKLTYGSQAGTTTAQTTQAQGYTQSAQVSP

RITFKPIAPSGQNATCTRYSYCNECMLDPDCGFCYKMNKSTVIDSSCVPVNKASTNEAAW

GRCENETKFKTEDIFWAYNFCPTPYSWTAQQGQTQYQTYYAPGMGPMPWTVNSEIYPLWA

RSTGNACSSGTNWTYNTYTSQTYQHTAEYQTYYGAYYQYAGYAATGQQYTYGCQPETKGK

KLEEIESLFDNRLCTCGTSDSDEGRYIEYIRVKGSNYHLSDNDASDVE

GTR14=GLUT14=SLC2A14= facilitated glucose transporter member 14, [Q8TDB8](https://www.uniprot.org/uniprot/Q8TDB8), 520aa


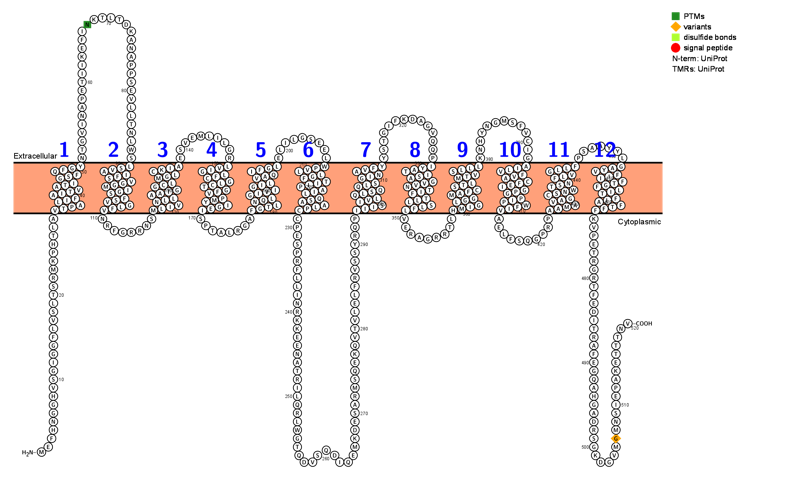


[Q8TDB8](https://www.uniprot.org/uniprot/Q8TDB8)| GTR14=GLUT14 Solute carrier family 2, facilitated glucose transporter member 14

MEFHNGGHVSGIGGFLVSLTSRMKPHTLAVTPALIFAITVATIGSFQFGYNTGVINAPET

IIKEFINKTLTDKANAPPSEVLLTNLWSLSVAIFSVGGMIGSFSVGLFVNRFGRRNSMLI

VNLLAATGGCLMGLCKIAESVEMLILGRLVIGLFCGLCTGFVPMYIGEISPTALRGAFGT

LNQLGIVIGILVAQIFGLELILGSEELWPVLLGFTILPAILQSAALPCCPESPRFLLINR

KKEENATRILQRLWGTQDVSQDIQEMKDESARMSQEKQVTVLELFRVSSYRQPIIISIVL

QLSQQLSGINAVFYYSTGIFKDAGVQQPIYATISAGVVNTIFTLLSLFLVERAGRRTLHM

IGLGGMAFCSTLMTVSLLLKNHYNGMSFVCIGAILVFVACFEIGPGPIPWFIVAELFSQG

PRPAAMAVAGCSNWTSNFLVGLLFPSAAYYLGAYVFIIFTGFLITFLAFTFFKVPETRGR

TFEDITRAFEGQAHGADRSGKDGVMGMNSIEPAKETTTNV


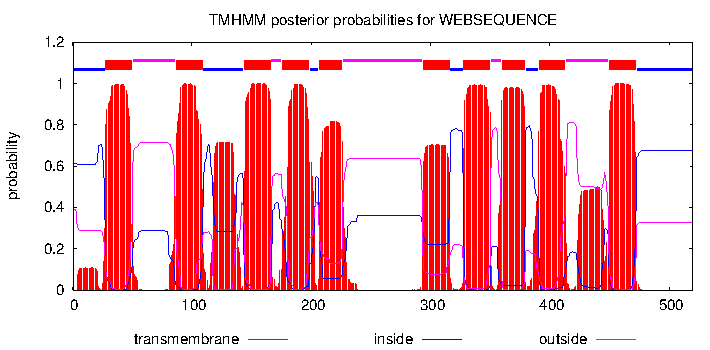

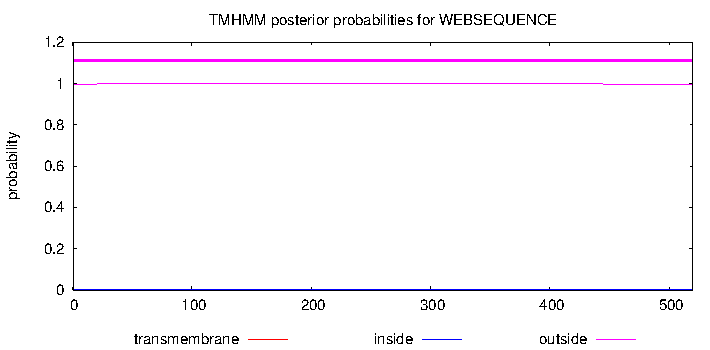


GLUT14^QTY^

MEFHNGGHVSGIGGFLVSLTSRMKPHTLATTPAQTYATTTATTGSYQYGYNTGVINAPET

IIKEFINKTLTDKANAPPSEVLLTNLWSQSTATYSTGGMTGSYSTGQYTNRFGRRNSMQT

TNQQAATGGCQMGQCKTAESVEMLILGRQTTGQYCGQCTGYTPMYTGETSPTALRGAYGT

QNQQGTTTGTQTAQTYGQELILGSEELWPTQQGFTTQPATQQSAAQPCCPESPRFLLINR

KKEENATRILQRLWGTQDVSQDIQEMKDESARMSQEKQVTVLELFRVSSYRQPTTTSTTQ

QQSQQQSGTNATYYYSTGIFKDAGVQQPTYATTSAGTTNTTYTQQSQYQVERAGRRTLHM

TGQGGMAYCSTQMTTSQQQKNHYNGMSFVCIGATQTYTACYETGPGPTPWYTTAELFSQG

PRPAAMATAGCSNWTSNYQTGQQYPSAAYYLGAYTYTTYTGYQTTYQAYTYYKVPETRGR

TFEDITRAFEGQAHGADRSGKDGVMGMNSIEPAKETTTNV
